# MEIS-1 level in unresectable hepatocellular carcinoma can predict the post-treatment outcomes of radiofrequency ablation

**DOI:** 10.18632/oncotarget.24165

**Published:** 2018-01-11

**Authors:** Hui Xie, Haipeng Yu, Shengtao Tian, Xueling Yang, Ximing Wang, Huaming Wang, Zhi Guo

**Affiliations:** ^1^ Department of Interventional Therapy, Tianjin Medical University Cancer Institute & Hospital, National Clinical Cancer Research Center, Key Laboratory of Cancer Prevention and Therapy, Tianjin 300070, PR China; ^2^ Department of interventional therapy, 302 Hospital of People's Liberation Army, Beijing 100039, PR China

**Keywords:** RFA, MEIS-1, unresectable HCC, prognosis

## Abstract

Radiofrequency ablation (RFA) is a local-ablative therapy for unresectable hepatocellular carcinoma (HCC). At present, there is no predictive marker for RFA treatment outcomes. This work aimed to valuate myeloid ecotropic viral integration site 1 (MEIS-1) in predicting post-RFA treatment outcomes of unresectable HCC patients. The time to progression (TTP) and overall survival (OS) of 81 HCC patients who received RFA treatment were measured. The protein level of MEIS-1 in tumor specimens was measured by western blot. The role of MEIS-1 in RFA-treating HCC *in vivo* growth nude mouse model was examined via PET/CT imaging. Higher level of MEIS-1 in tumor tissue is associated with better RFA treatment outcomes. The median TTP was 9.0 (95% confidence interval [CI]: 6.8–11.3) months in patients with high MEIS-1 expression (*n* = 43) versus 6.0 (95% CI: 4.6–7.4) months in patients with low MEIS-1 expression (*n* = 38). Moreover, in rodent HCC model we found overexpression of MEIS-1 enhanced the anti-tumor effect of RFA treatment. We conclude that high level of MEIS-1 expression predicts better RFA treatment outcome in HCC.

## INTRODUCTION

High proportion of hepatitis viruses infection makes China a heavily afflicted country of hepatocellular carcinomas (HCCs); effective HCC treatment approaches will relieve this urgent medical burden [1–3]. Normally HCC cannot be diagnosed until developed into advanced-stage which is not suitable for liver transplantation or surgical resection. There are limited treatment options available for advanced HCC patients, current HCC treatments have poor clinical outcomes and poor prognosis [4–6]. Radiofrequency ablation (RFA), an *in situ* ablative therapy which selectively destroys HCC tissues, is a promising treatment for advanced-stage HCC patients with cirrhosis and compromised liver function [7–10]. However, rapid or aggressive recurrence of HCCs after RFA treatment is a major obstacle [11]. It is urgent to study the mechanisms of the recurrence of HCCs after RFA treatment and identify predictive prognosis marker of HCC patients.

Transcription factor myeloid ecotropic viral integration site 1 (MEIS-1) is a member of the triple amino acid loop extension family, which are thought to play important roles in cell growth and differentiation during vertebrate embryogenesis [12]. MEIS-1 contains four functional domains: a N-terminal MEIS-A domain, a MEIS-B domain, a C-terminal transcription factor activity region, and a homeodomain, which links MEIS-B domain and the C-terminal region [13, 14]. Previously, MEIS-1 was reported as a positive tumor inducer [15–19]. However, MEIS-1 can also function as a negative regulator of cancers by inhibiting cell proliferation and inducing cell cycle arrest [20–21]. A previous study reported higher expression of MEIS-1 in healthy prostate tissues than in prostate carcinoma tissues, and concluded that MEIS-1 may serve as a predictive biomarker of prostate cancer prognosis [21]. Similar studies reported that MEIS-1 is a suppressor of non-small-cell lung cancer, esophageal squamous cell carcinomas and clear cell renal cell carcinomas [14, 22–25]. A recent paper reported that MEIS-1's expression level can predict treatment outcome in HCC patients [26].

In this study, we examined the predictive value of MEIS-1 expression in determining post-RFA treatment outcomes in HCC patients with advanced-stage disease. MEIS-1 may function as a negative regulator of HCC growth. Overexpression of MEIS-1 enhanced the efficiency of RFA’s anti-tumor effect on HCC cell proliferation *in vivo*. Thus, high level of MEIS-1 expression predicts better RFA treatment outcome in HCC.

## RESULTS

### Association of MEIS-1 protein level and RFA treatment outcomes

**Figure 1:**
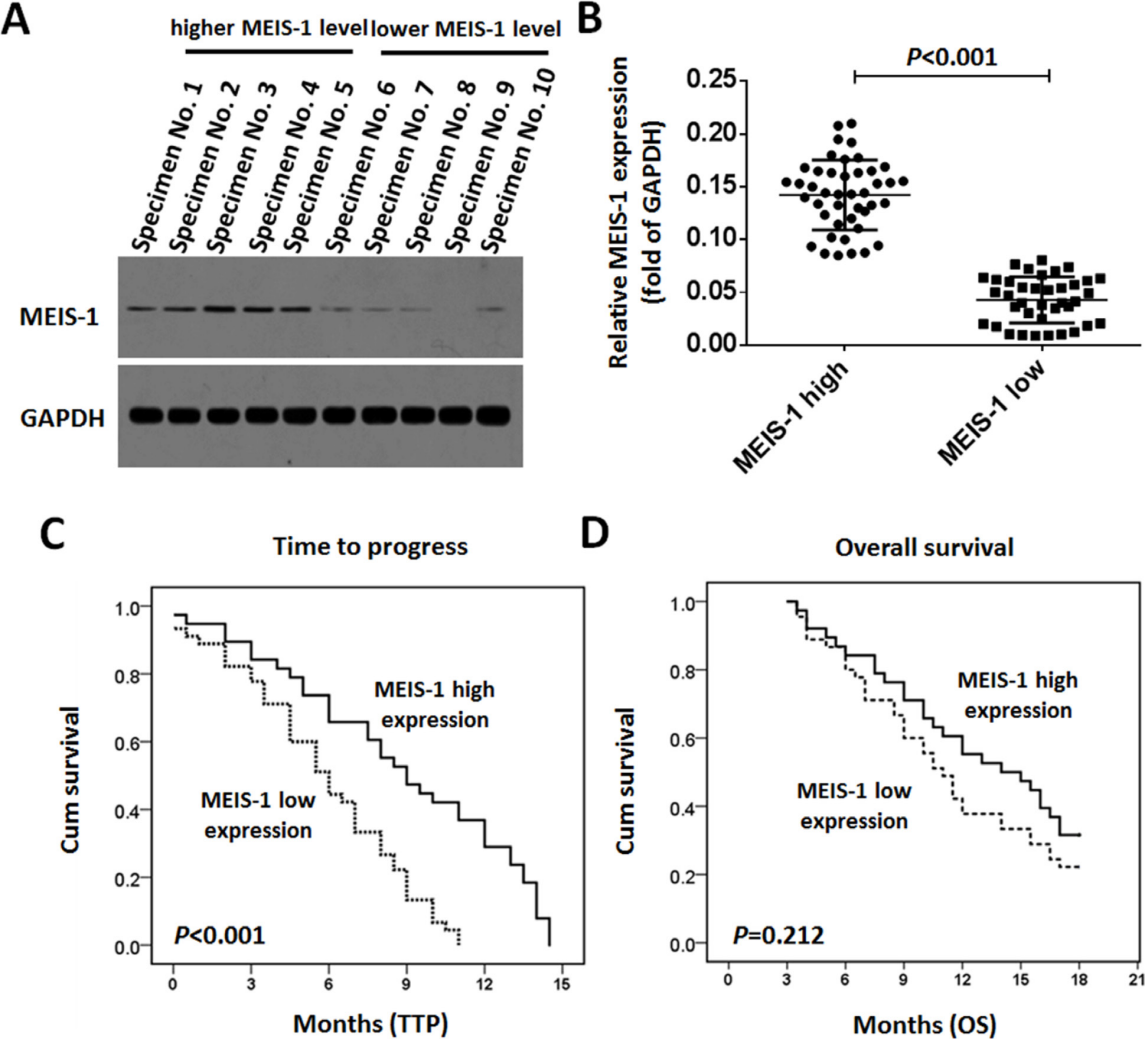
Expression of MEIS-1 and post-RFA outcomes of advanced HCCs The protein level of MEIS-1 in HCC clinical specimens was identified by a Western blot. (**A**) Representative photograph of MEIS-1 expression in clinical specimens. Tissue No. 1 to No. 5 indicated MEIS-1 high group, and No. 6 to No. 10 indicated the MEIS-1 low group. (**B**) Scatter diagram showing the intratumor levels and relative protein levels of MESI-1 in the two patient groups. (**C**) Kaplan–Meier estimates of the TTP in the two patient groups (*P* < 0.001). (**D**) D: Kaplan–Meier estimates of OS times in the two patient groups (*P* = 0.212).

The endogenous protein level of MEIS-1 in clinical specimens was detected by western blot. The results of the quantitative analysis of the western blots are shown in Figure 1A. According to the median value of intratumoral MEIS-1's protein expression, the 81 patients were divided into two groups: those with low MEIS-1 expression (*n* = 38) and those with high MEIS-1 expression (*n* = 43), as shown in Figure 1B.

As shown in Figures 1C and 1D, and Table 1, the median post-RFA TTP is 9.0 (95% confidence interval [CI]: 6.8–11.3) months in the high MEIS-1 expression group, whereas it is 6.0 (95% CI: 4.6–7.4) months in the low MEIS-1 expression group (log-rank *P* < 0.001, Figure 1C). However, the difference in the median OS of the two groups seem obvious but is not significant (log-rank *P* = 0.212, Figure 1D). As shown in Table 1, patients with high MEIS-1 expression had higher CER and DCR (39.53% vs. 15.79%, *P* = 0.031 for CER; 67.44% vs. 42.10%, *P* = 0.022 for DCR) compared with those with low MEIS-1 expression (Table 1).

**Table 1:** MEIS-1’s protein level and outcomes of post-RFA

	MEIS-1 protein level		*P*
	High (*n* = 43)	Low (*n* = 38)	
TTP	9.0 (M)	6.0 (M)	< 0.001
	6.8–11.3 (M)	4.6–7.4 (M)	
OS	14.0 (M)	11.0 (M)	0.212
	8.7–19.3 (M)	9.4–12.6 (M)	
Overall response rate (CR + PR)	17 (39.53%)	6 (15.79%)	0.031
Disease control rate (CR + PR + SD)	29 (67.44%)	16 (42.10%)	0.022

TTP: time to progress; OS: overall survival; PR: partial remission; CR: complete remission; SD: stable of disease; M: months.

### MEIS-1 enhanced RFA-induced inhibition of HCC cells *in vivo* growth

**Figure 2:**
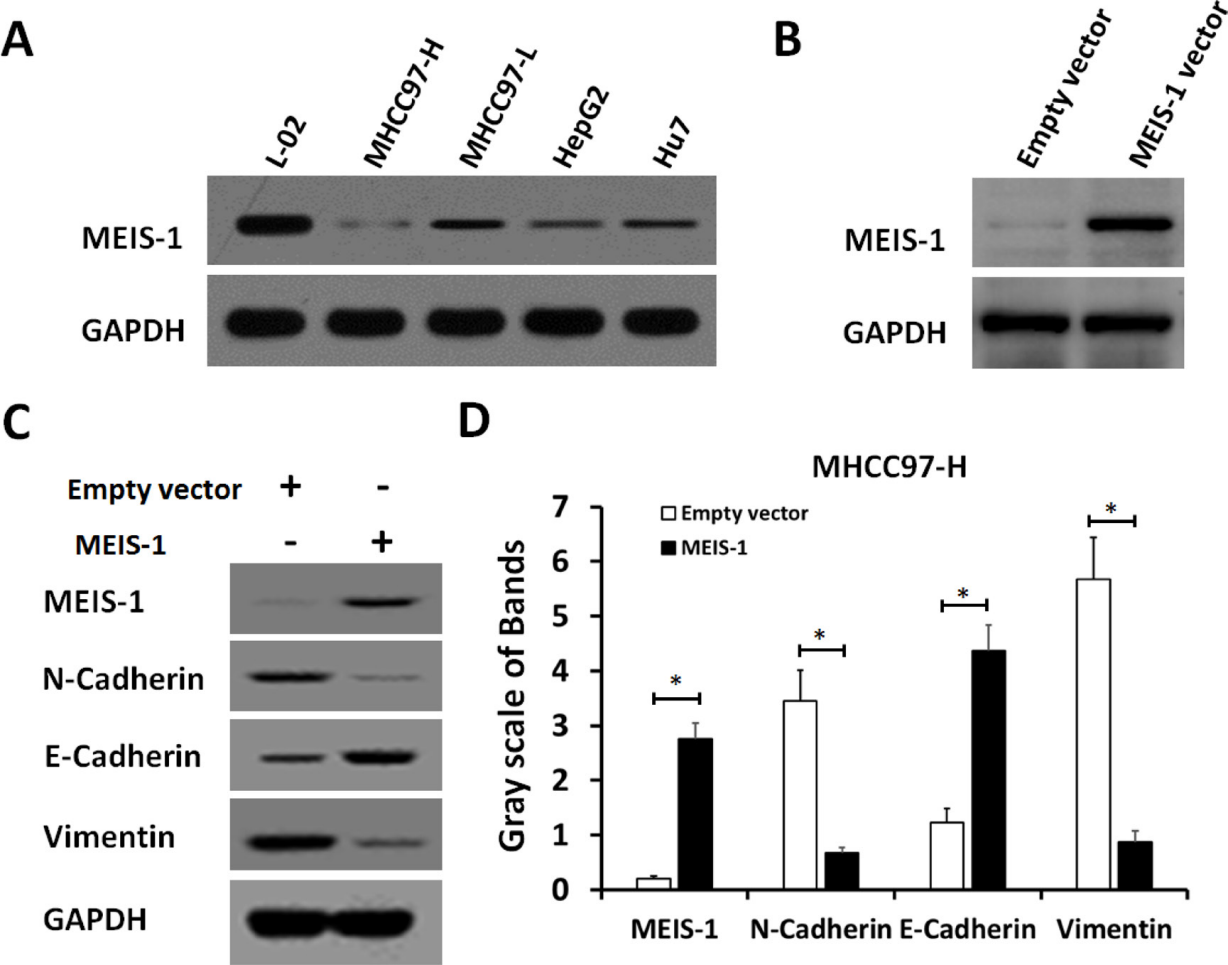
MEIS-1 inhibits the epithelial-mesenchymal transition (EMT) of MHCC97-H cells (**A**) The protein level of MESI-1 was identified in a hepatic nontumor cell line (L-02) and HCC cells (HepG2, MHCC97-H, MHCC97-L, and Hu7). (**B**) MHCC97-H cells infected with an empty vector or MEIS-1 were harvested and analyzed by a western blot. (**C**, **D**) The Protein level of MEIS-1, E-cadherin, N-Cadherin or Vimentin was identified by its antibody.

To validate whether MEIS-1 intrinsicly expression affects tumor growth, we next established HCC cell lines with low or high MEIS-1 expression level. We first selected a HCC cell line MHCC97-H which has very low endogenous MEIS-1 expression (Figure 2A), MEIS-1 was overexpressed *via* infection of its adenovirus-vectors in MHCC97-H cells (Figure 2B). We found the overexpression of MEIS-1 increased the expression of E-cadherin, an epithelial marker, and decreased the protein level of N-cadherin and Vimentin, two mesenchymal markers (Figure 2C and 2D). Therefore, MEIS-1 inhibits the epithelial-mesenchymal transition of MHCC97-H cells.

**Figure 3:**
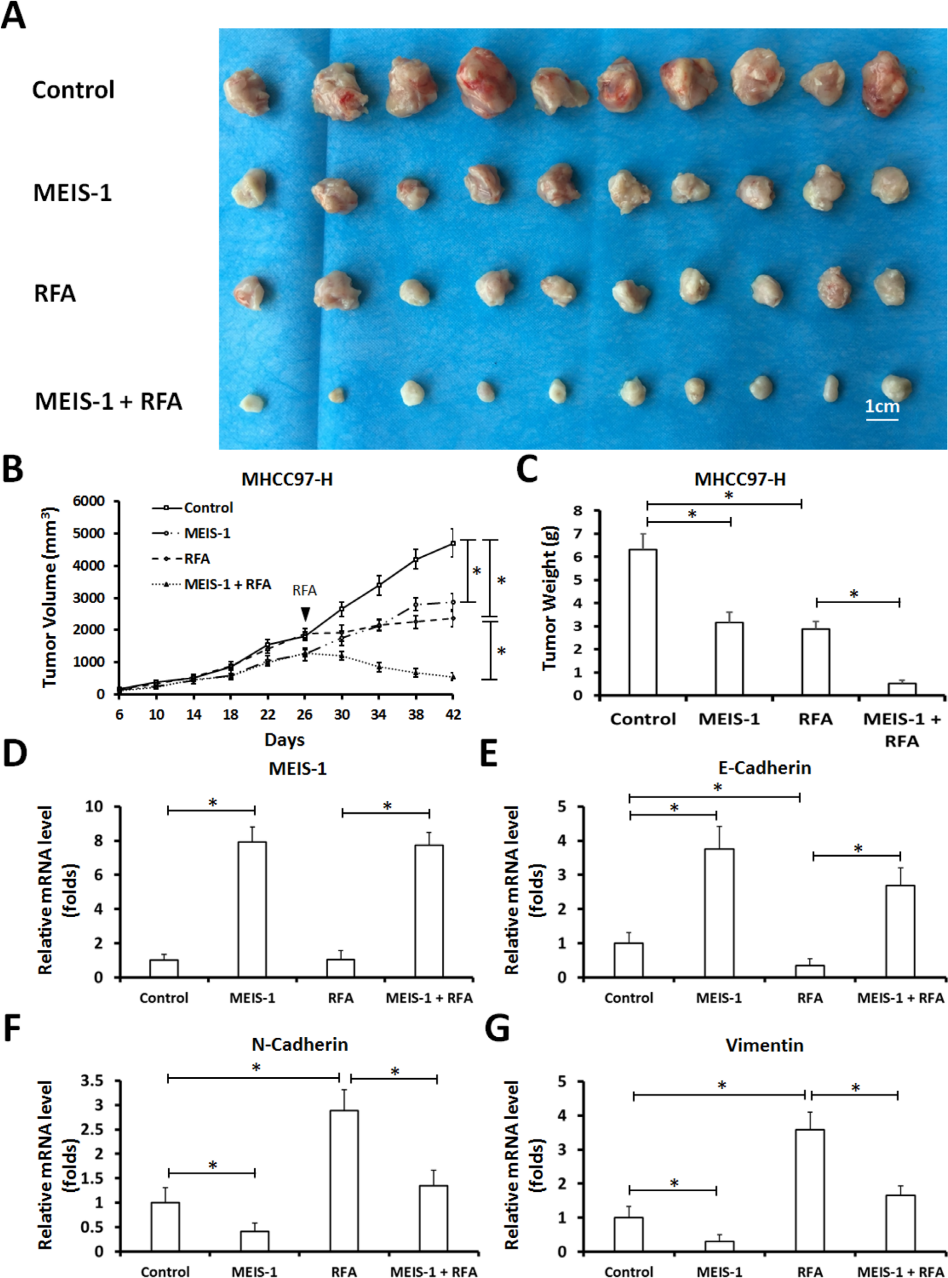
Impact of overexpression of MEIS-1 on subcutaneous growth of HCC cells in RFA-treated nude mice (**A**) MHCC97-H cells infected with an empty vector or MEIS-1 were injected into nude mice. When the tumoral volume reached 1000–1200 mm^3^, RFA was performed. Tumoral growth was defined as the tumoral volume (**B**) and tumoral weight (**C**). The relative mRNA level of MEIS-1 (**D**), E-cadherin (**E**), N-Cadherin (**F**) or Vimentin (**G**) in tumors was shown as mean mean ± SD. ^*^P < 0.05.

Next, control MHCC97-H cells or MEIS-1 overexpressing MHCC97-H cells were injected into nude mice subcutaneously. We observed that overexpression of MEIS-1 reduced tumor growth, and the RFA treatment resulted in shrinkage of the tumors (Figure 3A–3C). Moreover, RFA induced the EMT process of HCC tumors, MEIS-1 inhibited the EMT induced by RFA (Figure 3D–3G). Thus, overexpression of MEIS-1 enhances the anti-tumor effect of RFA treatment.

**Figure 4:**
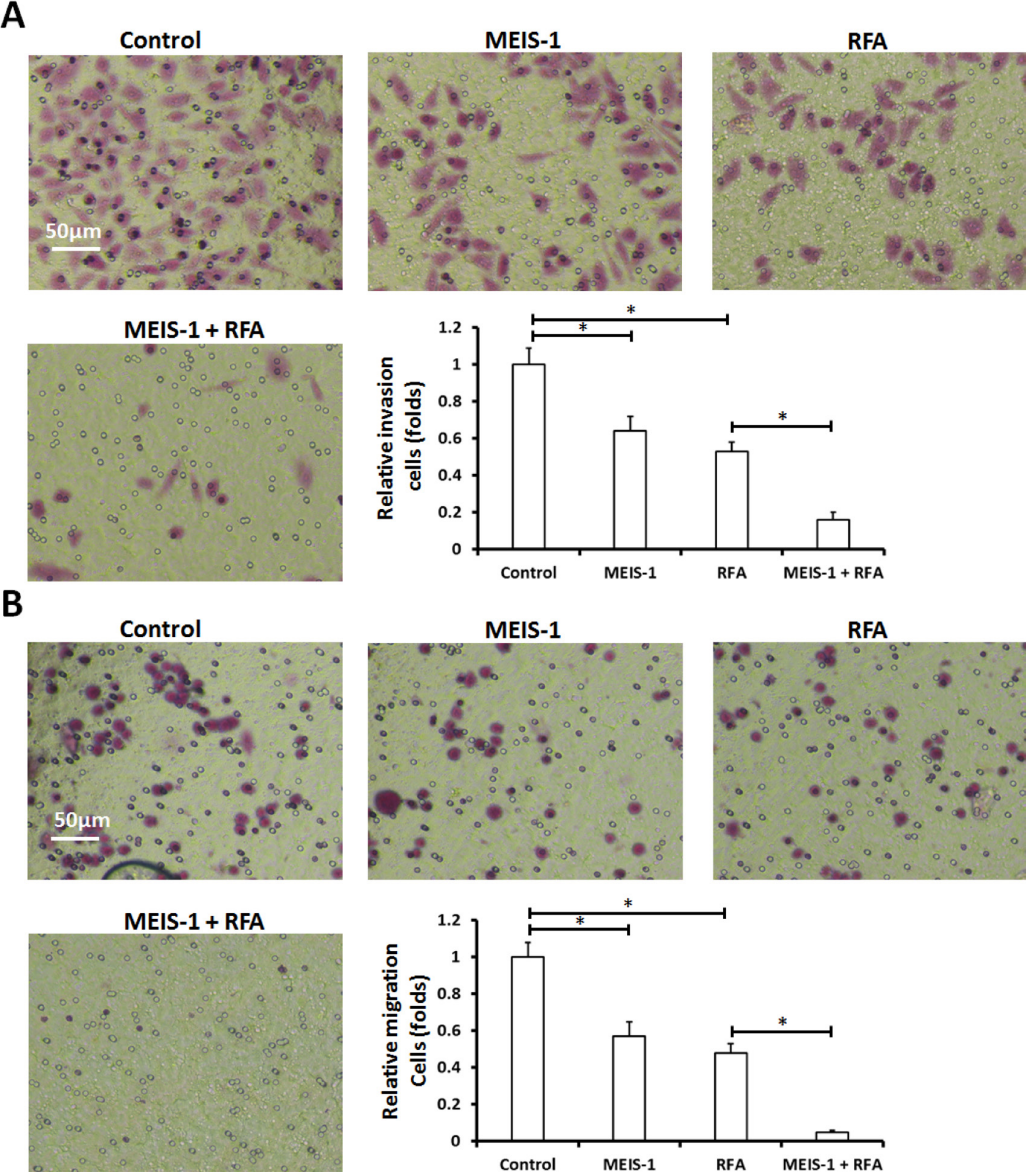
*In vitro* invasion or migration of cells separated from subcutaneous tumors The subcutaneous tumors described in Figure 3 were harvested. Then, single cells were separated from the tumors and analyzed using a transwell experiment. The *in vitro* invasion (**A**) or migration (**B**) was shown as representative photographs or mean ± SD. ^*^P < 0.05

Next, HCC cells were isolated from the subcutaneous tumors (Figure 3) that had been treated with RFA or infected with vectors; transwell assays (*in vitro* invasion or migration) and intrahepatic growth assay (injected into the liver lobe) were performed. As shown by the results of the soft agar assay, overexpression of MEIS-1 or RFA attenuated *in vitro* invasion (Figure 4A) and migration (Figure 4B) of HCC cells.

**Figure 5:**
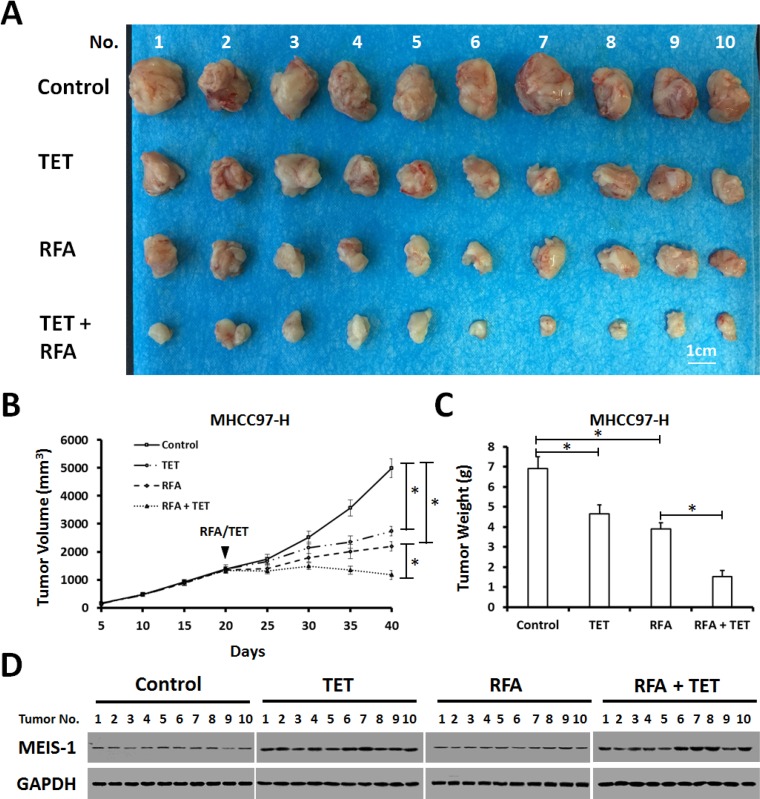
Impact of overexpression of MEIS-1 on subcutaneous growth of HCC cells in RFA-treated nude mice (**A**) MHCC97-H cells infected with an empty vector or TET-on-MEIS-1 were injected into nude mice. When the tumoral volume reached 1000–1200 mm^3^, RFA was performed. Next, mice were received solvent control or tetracycline per day. The tumor growth was defined as the tumoral volume (**B**) and tumoral weight (**C**). (**D**) The expression of MEIS-1 in tumor tissues was identified by western blot *via* its antibody.

To confirm the results in *in vivo* tumor growth, the effect of MEIS-1 on RFA was examined by inducing expression model. MHCC97-H cells infected with control virus or TET-on virus which express MEIS-1 were injected into nude mice subcutaneously. When the volumes of subcutaneous tumors reach 1200mm^3^, the RFA was performed. Next, mice received solvent control or tetracycline (Tetracycline does not affect HCC tumor growth, shown in Supplementary Figure 1). As expected, tetracycline treatment induced the overexpression of MEIS-1 and reduced tumor growth (Figure 5); and the RFA treatment resulted in tumor size reduction (Figure 5).

**Figure 6:**
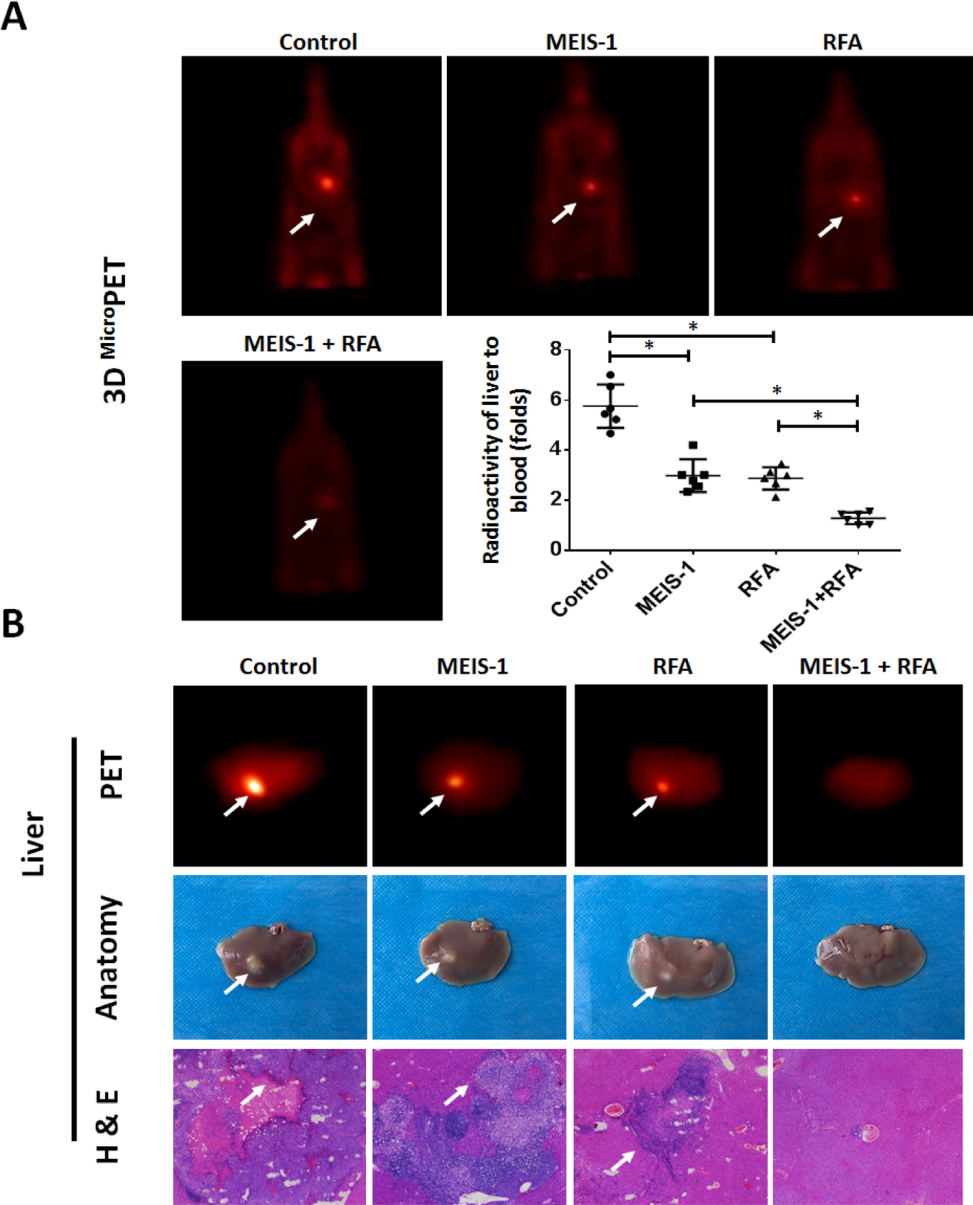
Intrahepatic growth of cells separated from subcutaneous tumors The subcutaneous tumors described in Figure 3 were harvested. Then, single cells were separated from the tumors and injected into the right lobe of the liver. After 4–8 weeks, ^18^F-FDG/PET images (*n* = 6) were obtained (**A**). (**B**) The results of the PET/CT were confirmed by the radioactivity of ablated livers and H&E staining. The arrows indicate intrahepatic tumor nodules. ^*^P < 0.05.

Moreover, we validated our hypothesis in *in situ* HCC cell growth model in which the intraliver tumors were measured by PET/CT. As shown in Figure 6A, RFA treatment decreased the intrahepatic growth of HCC cells. Overexpression of MEIS-1 enhanced the inhibitory effect of RFA on HCC cells’ nodule formation in liver (Figure 6A). The liver-to-blood radioactive data, tumor foci in the whole liver, and PET imaging of the whole liver confirmed the PET imaging of whole animals (Figure 6B). Thus, overexpression of MEIS-1 enhanced the anti-tumor effect of RFA treatment in *in situ* HCC.

## DISCUSSION

It is well known that advanced HCC patients have a poor prognosis due to the ineffectiveness of systemic chemotherapy and local radiation therapies [27, 28]. Recent studies reported that Sorafenib, an oral kinase inhibitor, improved the OS and life quality of HCC patients [29–31]. However, intrinsic or acquired Sorafenib resistance in tumor tissue restricts the effect of this promising approach [29–31]. On the other hand, local-ablative therapy has becoming a new hope for patients suffering from unresectable HCC. RFA is widely used to treat advanced HCC patients, as it presents better therapeutic outcomes compared to other local ablative methods [32]. Nevertheless, there is not an effective indicator of post-RFA prognosis.

MEIS-1 play a critical role in several important physiological processes, such as organ development and stem cell differentiation [33, 34]. It also participates in the development and progression of cancers. For example, aberrant expression of MEIS-1 promoted the development of acute myeloid leukemia [15–19, 35]. However, MEIS-1 has been reported as a negative regulator of some other cancers, such as non-small-cell lung cancer, esophageal squamous cell carcinomas, clear cell renal cell carcinomas and prostate cancer [22–25]. In these studies, the authors proposed that MEIS-1 inhibits tumor cell proliferation and induces cell cycle arrest [22–25]. In the present study, the patients with high MEIS-1 expression show better post-RFA outcomes than those with low MEIS-1 expression. The median TTP was also longer in the high MEIS-1 group than in the low MEIS-1 group. Furthermore, the CER and CDR of the patients with high MEIS-1 expression were better than those of the patients with low MEIS-1 expression. These results indicated that MEIS-1’s expression level indicates the anti-tumor effect and prognosis of RFA therapy.

RFA is one of the most suitable treatment option for advanced-stage HCC, particularly when a patient’s liver functional reserve precludes radiotherapy. However, HCC can recur after RFA, and the phenotype, *e.g.* Epithelial-mesenchymal transition, of the tumor cells often changes [36]. Previous research suggested that incomplete ablation in RFA may induce cellular stress and lead to pathological changes [37, 38]. Zhu et al. suggested that overexpression of MEIS-1 inhibits the EMT process and decreases the expression of pro-survival genes in clear cell renal cell carcinomas [25]. Yasui et al. [26] indicated that MEIS-1 may be a suppressor of TGFβ signaling pathway, which is one of the foremost mediators of EMT. TGFβ/Smad signaling pathway plays a crucial role in tumorigenesis and tumor development, included HCC [39, 40]. It has been previously reported that TGFβ/Smad can be a tumor suppressor and mediate the expression of tumor suppressor p15 and p21 [41–44]. Zhou et al. showed that Smad3 can sensitize HCC cells to cisplatin treatment by repressing Akt phosphorylation [44]. At same time, other papers also reported that TGFβ/Smad pathway may facilitate tumor growth [45–47]. It is well known that TGFβ/Smad pathway plays a central role in EMT process [48].

In the present study, we established a subcutaneous tumor model and then performed RFA at 65–70°C for 3–5 min to attenuate the growth of HCCs. Overexpression of MEIS-1 enhanced the anti-tumor effect of RFA. In the future, in addition to RFA treatment, anti-tumor agents could be injected into tumors to mimic TACE. In addition, intrahepatic growth of HCC cells in nude mice could be achieved by directly ablate the lesion in liver using abdominal surgeries in mice. Local ablation of intrahepatic HCC by RFA could also be performed in some larger animals, *e.g.* immunodeficient rat, guided by small animal molecular imaging system, *e.g.* small animal ultrasound systems.

## MATERIALS AND METHODS

### Patients

This prospective study consisted of 81 consecutive patients who underwent RFA between April 2014 and May 2016, and the baseline clinical data of patients with advanced HCC were shown in Table 2. The inclusion criteria were as follows [7, 49–51]: (A) a diagnosis of HCC and stage B or C unresectable cancer; (B) the presence of portal hypertension and Child’s class A or B cirrhosis according to endoscopy or imaging; (C) a life expectancy of at least 12 weeks according to the clinical presentation; (D) Eastern Cooperative Oncology Group performance status 0, 1, or 2; and (E) histological grade of HCC differentiation classed as well differentiated, intermediately differentiated, or poorly differentiated according to the criteria of Edmondson. The collection of the HCC specimens and study protocol were approved by the Ethics Committee of the 302nd Hospital, and informed consent was obtained from all the patients.

**Table 2:** Baseline clinical data of 81 patients with advanced HCC

Presentation	Case (%)
Median age, yr (range)	49 (28–67)
Gender, male (%)	68 (83.95%)
Aetiology (%)	
HBV positive	71 (87.65%)
HCV positive	10 (12.35%)
ECOG PS (%)	
0	31 (38.27%)
1	46 (56.79%)
2	4 (4.94%)
AFP (%)	
Normal	17 (20.99%)
Elevated	64 (79.01%)
extrahepatic metastasis (%)	32 (39.50%)
LN metastasis (%)	39 (48.14%)
Portal vein invasion (%)	43 (53.08%)
Chilg-Pugh (%)	
A	69 (85.18%)
B	12 (14.82%)
Median size of index tumor, cm (range)	1.4 (1–3.0)
Median number of index tumors	2 (1–3)
Differentiation	
Well	10 (12.34%)
Moderately	44 (54.32%)
Poorly	27 (33.33%)
Prior local therapy (%)	None

None of the HCC patients had received any prior treatment. Clinical specimens were obtained by a puncture biopsy using a coaxial needle (Cat. no.: MCXS1815BP, RITA Company, Crystal Lake, IL, USA) immediately after the RFA treatment. The primary endpoint was the time to progression (TTP) post-RFA, and the secondary endpoint was overall survival (OS). The association of the protein expression of MEIS-1 with post-RFA OS and TTP was assessed in the same cohort of patients. The efficacy of RFA was based on the clinical efficacy response (CER)/overall response rate or disease-control rate (DCR), in accordance with the protocols described in our previous work [7, 51]. The overall response rate was defined as: complete response (CR) + partial response (PR) [7, 51]. The disease-control ate was defined as: CR + PR + stable disease (SD) [7, 51].

### Cell culture and Western blot analysis

A hepatic nontumor cell line, L-02, and HCC cell lines (HepG2, MHCC97-H, MHCC97-L, and Hu7) were cultured in Dulbecco’s modified Eagle medium (DMEM) with 10% fetal bovine serum at 37° C in an atmosphere of 5% CO_2_ [51, 52]. Tetracycline (Cat. no.: S2574) was purchased from Sellck Corporation, Houston, Texas, USA. An empty vector adenovirus and MEIS-1 were purchased from Vigene Company (Jinan, Shandong, China). A TET-on Lentivirus of MEIS-1 was generated and prepared by Vigene Company (Jinan, Shandong, China). Cells were harvested for Western blot analysis. Total protein was extracted from cells or clinical specimens and subjected to sodium dodecyl sulfate polyacrylamide gel electrophoresis. The proteins were then transferred to a polyvinylidene fluoride film. Next, the blots were blocked by 5% bovine serum albumin and incubated with the primary antibody anti-MEIS-1 immunoglobulin G (IgG) (Cat. no.: sc-101850, Santa Cruz, USA) in a 1:2000 dilution, anti-E-Cadherin immunoglobulin G (IgG) (Cat. no.: sc-71009, Santa Cruz, USA) in a 1:5000 dilution, anti-N-Cadherin immunoglobulin G (IgG) (Cat. no.: sc-59987, Santa Cruz, USA) in a 1:1000 dilution, anti-Vimentin immunoglobulin G (IgG) (Cat. no.: sc-73258, Santa Cruz, USA) in a 1:1000 dilution or anti-glyceraldehyde-3-phosphate dehydrogenase (GAPDH) IgG (Cat. no.: sc-47724, Santa Cruz, USA) in a 1:5000 dilution. After washing three times in tris-buffered saline, the blots were incubated with a secondary antibody (1:5000 dilution) and developed by the addition of enhanced chemiluminescence reagents (Qiangen, Beijing, China) and X-ray film exposure. The quantitative protein level of MEIS-1 was determined by gray-scanning analysis using Alpha Innotech analysis software (San Leandro, CA, USA). The protein level of MEIS-1 was normalized to that of a loading control (GAPDH). What was calculated using the following formula: gray scale of MEIS-1 band)/(gray scale of GAPDH).

### RFA treatment

A multipolar 15 cm-long RFA needle (Cat. no.: UniBlate 700-103597, RITA Company, Crystal Lake, IL, USA), with a maximum ablation diameter of 5 cm was used in all cases. Percutaneous RFA was performed using a radiofrequency therapy instrument (Cat. no.: RITA Model 1500X, RITA Company, Crystal Lake, IL, USA), with guidance provided by computed tomography (CT) scanning. The RFA needle was inserted in the direction of the tumor, and CT scanning was used to confirm the position of the RFA needle. Ablation started once the needle reached the required position. The ablation time was 15–20 min, and the temperature setting was 105°C.

### Animal experiments

**Figure 7:**
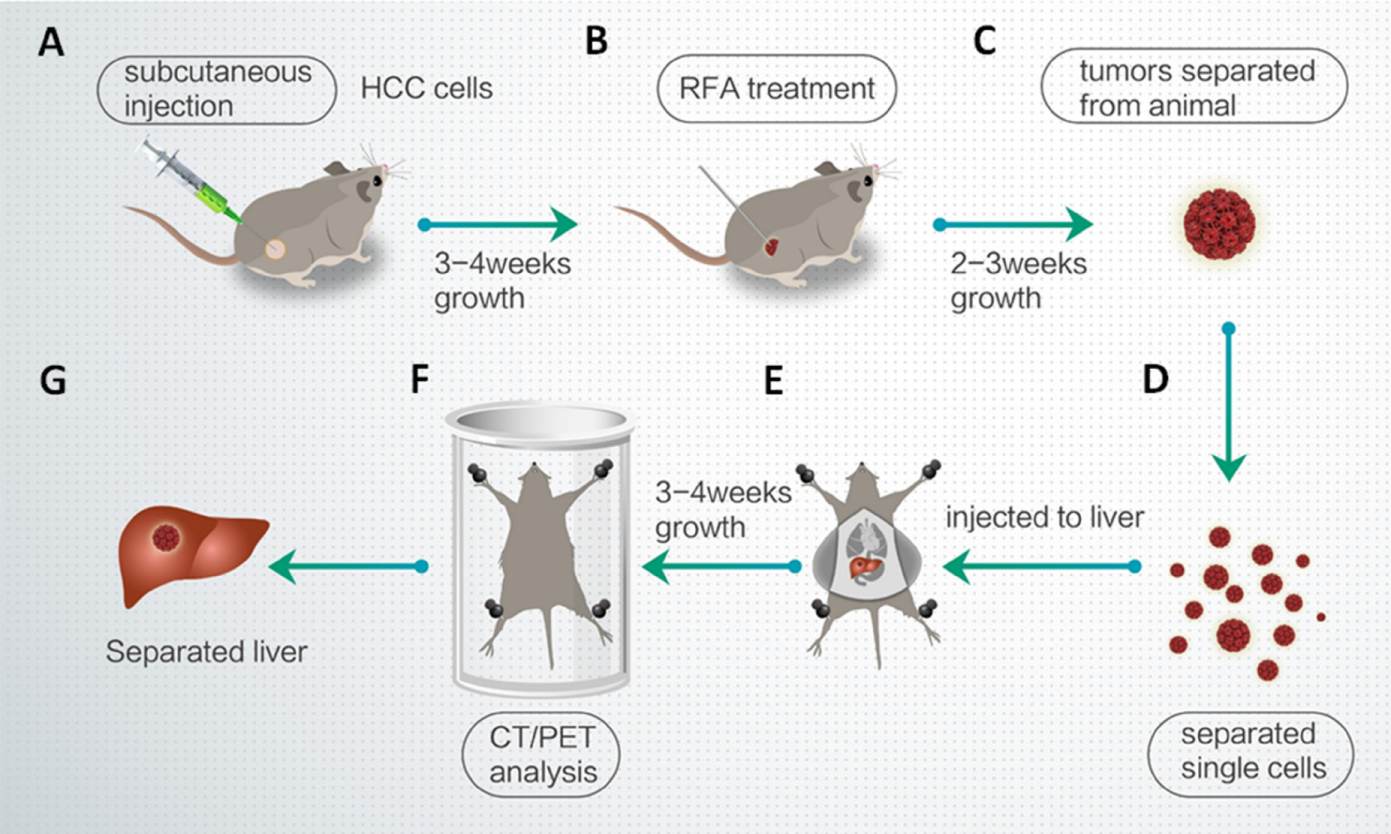
Workflow of the animal experiments (**A**) MHCC97-H cells were injected into nude mice. (**B**) After 3-4 weeks, the subcutaneous tumors were treated with RFA. (**C**) After 2–3 weeks, the tumors were removed. (**D**) Single cells were separated from the tumors by tissue-grinding and sieve-filtrating. (**E**) The cells were then injected into the right lobe of the liver. (**F**) After 3–4 weeks, intrahepatic HCCs were identified by CT/PET. (**G**) The liver was anatomized and separated to show the intrahepatic lesions/intrahepatic nodules formed by HCC cells.

All the animal experiments were reviewed and approved by the Institutional Animal Care and Use Committee of the 302nd Hospital, People’s Liberation Army of China. Nude SCID (severe combined immune deficiency) mice aged 4–6 weeks were purchased from Si-Bei-Fu Biotechnology Corporation, Beijing China. Figure 7 shows the workflow of the animal experiments.

To produce a subcutaneous tumor model [53–55], MHCC97-H cells infected with a control or MEIS-1 virus vector were injected into nude mice (1 × 10^6^ cells per animal). After 4–6 weeks (26 days), the tumoral volume had reached almost 1200 mm^3^. RFA of the subcutaneous tumors was performed using a thyroid ablation needle (Cat. no.: UniBlate 700-103587 17G, RITA Company). The ablation time was 3–5 min, and the temperature setting was 65–70° C. After the RFA treatment, the tumoral volume was calculated every 4 days using the following formula: width^2^ × length/2. Tumors were harvested 16 days after the RFA treatment, and their weights were measured.

To produce a liver *in situ* tumor model [56–58], HCC cells were separated from subcutaneous tumors formed by MHCC97-H cells and directly inoculated into the right lobe of the liver (1 × 10^5^ cells per animal). After 4–8 weeks, nude mice were injected intravenously with 100 μCi of 18F radio-labeled fluorodeoxyglucose (^18^F-FDG), and the animals were examined using a positron emission tomography/computed tomography (PET/CT) scanner (Philips Corp., Holland). Two-minute CT and 10-min PET scans were performed 45 min after the FDG injection. A NaI (Tl) well counter (China Atom Corp., Beijing China) was used to measure the radioactivity of organs (liver) and blood.

### qPCR

The qPCR (Quantitative reverse-transcription PCR) was performed following the methods descripted by Feng et al., 2015 [59]. Briefly, RNA samples were extracted from subcutaneous tumor by PARISTM Kit (Applied Biosystems, Foster City, CA, USA) and reverse-transcribed to cDNA by a Multiscribe™ Reverse Transcriptase kit (Applied Biosystems, Foster City, CA, USA) according to the manufacturer’s instructions. The mRNAs level of MEIS-1, E-Cadherin, N-Cadherin or Vimentin was examined by qPCR. The sequences of the primers used were presented in Supplementary Table 1 [60].

### Transwell analysis

MHCC97-H cells were injected into nude mice to produce subcutaneous tumors. Subsequently, the tumors were divided into an RFA-treatment group and a non-RFA treatment group. Next, single cells (3000 per well) were separated from the subcutaneous tumors and were analyzed by transwell assays performed in 24-well plates chamber (Corning, Lowell, MA, USA) fitted with a polyethylene terephthalate filter membrane with 8-μm pores. The invasion cells or migration cells were measured following the methods descripted by Zhou et al. and Yang et al. [61, 62].

### Statistical analysis

All the statistical analyses were performed using SPSS software (version 16.0). Data are presented as the median and range. The OS rate was estimated by the Kaplan–Meier method, and groups were compared by the log-rank test. Pearson’s Chi-square test was used to test the difference between the CER and DCR of the MEIS-1 high and MEIS-1 low groups. In all the analyses, a *P*-value < 0.05 was considered statistically significant.

## CONCLUSIONS

The endogenous level of MEIS-1 seems to be associated with post-RFA treatment outcomes. The median post-RFA TTP of patients with a low MEIS-1 expression level was significantly shorter than patients with a high MEIS-1 expression level. To validate this, we used rodent tumor growth model. Overexpression of MEIS-1 enhanced the tumor rejection effect of RFA in preventing subcutaneous growth of HCC cells. The findings indicate that MEIS-1 and RFA may exert a synergetic effect, resulting in a reduction in the tumor volume. Our data suggest that the expression level of MEIS-1 may serve as a clinical predictor of post-RFA treatment outcomes in HCC patients.

## SUPPLEMENTARY MATERIALS TABLE AND FIGURE



## Abbreviations

MEIS-1: myeloid ecotropic viral integration site 1
RFA: radiofrequency ablation
HCC: hepatocellular carcinoma
TTP: progression
OS: overall survival
TALE: Triple amino acid loop extension
HD: homeodomain
AML: acute myeloid leukemia
CER: Clinical efficacy response
CR: complete response
PR: partial response
DCR: Disease-control rate
SD: stable disease
PVDF: Polyvinylidene Fluoride
18F-FDG: 18F radio-labeled fluorodeoxyglucose
NSCLC: non-small-cell lung cancer
ESCC: esophageal squamous cell carcinoma
ccRCC: clear cell renal cell carcinoma
PC: prostate cancer
EMT: Epithelial-mesenchymal transition.
