# Supplementary material for: MEIS-1 level in unresectable hepatocellular carcinoma can predict the post-treatment outcomes of radiofrequency ablation
Source: Oncotarget. 2018 Jan 11;9(20):15252–65. doi: 10.18632/oncotarget.24165 (PMC5880601; doi:10.18632/oncotarget.24165)
Supplement: Supplementary file 1 [file oncotarget-09-15252-s001.pdf]

MEIS-1 level in unresectable hepatocellular carcinoma can predict the post-treatment outcomes of radiofrequency ablation

SUPPLEMENTARY MATERIALS

Supplementary Table 1: The primers used in this work

Genes	Primer sequence (5′–3′)
MEIS-1	Forward: 5′-TCCCAA AGTAGCCACCAATATC-3′
	Reverse: 5′-CTGTATCTGTGCCAAC TGCTT-3′
E-cadherin	Forward: 5′-AAGGCACGCCTGTCGAAGCA-3′
	Reverse: 5′-ACGTTGTCCCGGGTGTCATCCT-3′
N-cadherin	Forward: 5′-TGCGCGTGAAGGTTTGCCAGT-3′
	Reverse: 5′-TGGCGTTCTTTATCCCGGCGT-3′
Vimentin	Forward: 5′-ACCGCACACAGCAAGGCGAT-3′
	Reverse: 5′-CGATTGAGGGCTCCTAGCGGTT-3′

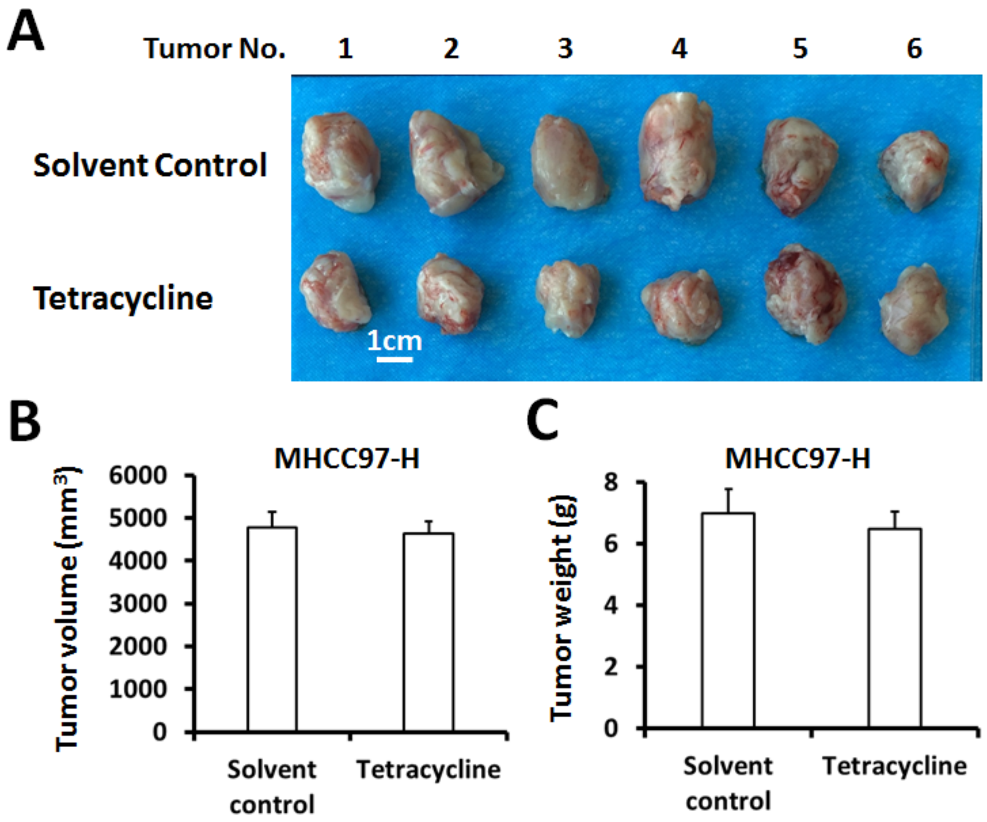

Supplementary Figure 1: The effect of tetracycline on HCC cells' subcutaneous growth. (**A**) MHCC97-H cells were seeded in nude mice. When the tumoral volume reached 1000-1200 mm^3, Mice were received solvent control or tetracycline per day. After 2-4 weeks' growth, tumoral growth was defined as the tumoral volume (**B**) and tumoral weight (**C**).
